# What is the Need for Prostatic Biomarkers in Prostate Cancer Management?

**DOI:** 10.1007/s11934-015-0545-3

**Published:** 2015-08-13

**Authors:** Martin Spahn, Silvan Boxler, Steven Joniau, Marco Moschini, Bertrand Tombal, R. Jeffrey Karnes

**Affiliations:** Department of Urology, Inselspital, Bern University Hospital, University of Bern, Anna Seiler Haus, CH-3010 Bern, Switzerland; Urology, Department of Development and Regeneration, University Hospitals Leuven, Herestraat 49, 3000 Leuven, Belgium; Department of Urology, Cliniques universitaires Saint Luc, Avenue Hippocrate, 10, B-1200 Brussels, Belgium; Department of Urology, Mayo Clinic, 200 1st St. SW, Rochester, MN 55905 USA

**Keywords:** Prostate cancer, Genetics, Microenvironment, Treatment, Biomarkers

## Abstract

Discriminating patients with a low risk of progression from those with lethal prostate cancer is one of the main challenges in prostate cancer management. Indeed, such discrimination is essential if we aim to avoid overtreatment in men with indolent disease and to improve survival in those men with lethal disease. We are reporting on the current literature on such prognostic tools that are now available, their clinical role and their limitations in individualizing care. There is an urgent need to incorporate such genomic tools into new platform-based clinical trial structures to further develop and validate prognostic and predictive biomarkers and provide prostate cancer patients with an effective and cost-efficient access to new drugs in the setting of personalized treatment.

## Introduction

In Europe, over 417,000 men were diagnosed with prostate cancer in 2012. Some patients will have highly aggressive disease that might metastasize early and ultimately prove fatal. In Europe, more than 90,000 men die of prostate cancer every year, that is less than one out of five patients [1]. The majority of the patients will have slow growing relatively harmless tumors that will not threaten their health during lifetime and can be appropriately managed with active surveillance or local treatment. For those developing lethal disease, there has been a major advance in treatment with the introduction of seven systemic treatments in addition to androgen deprivation therapy that has been the sole systemic treatment for the last 60 years. Unfortunately, their use has been constrained to patients who have failed local and hormonal treatment thus conferring a very limited benefit of a few months. To increase survival, it is crucial to move this drug earlier in the disease. But the accurate risk stratification becomes compulsory to avoid overtreatment of indolent disease and concentrate on men with lethal tumors. Compared to diagnostic standards applied in other cancers, the evaluation of prostate cancer aggressiveness is still archaic. Initial evaluation and treatment allocation are based exclusively on the combination of PSA value, histological grading, and clinic-radiological TNM staging. In this context, many studies have focused on the molecular basis of prostate cancer and highlighted several alterations in well-characterized cancer pathways, the androgen/androgen receptor (AR) signaling, gene fusions or mutations directly related to gene expression, and chromatin regulation. Promising genomic tools have been recently developed to predict an individual patient’s prognosis or response to secondary hormonal therapy. However, prostate cancer is a genetically heterogeneous disease and despite the impressive progress in cataloging genomic alterations in prostate cancer, the prognostic significance of the majority of these changes remains unclear and complicates risk stratification and selection of management strategies.

In this review, the focus is on currently available tissue-based genomic markers, their role in daily clinical practice and the needs for clinical trials to prove the value of these markers in precision prostate cancer care.

## Genetic Pathways in Prostate Cancer

Several genomic abnormalities were described in relation to prostate cancer development and progression. The Catalogue of Somatic Mutations in Cancer (COSMIC) database, an online database of somatically acquired mutations found in human cancers, displays data from different tumors including several hundreds of prostate cancer samples (http://cancer.sanger.ac.uk/cosmic) [2]. Among the top 20 most frequently altered genes in prostate cancer are several, well-known genes involved in tumor development and progression. The most frequently altered genes are TP53 (10 %), PTEN (8 %), SPOP (7 %), KRAS (5 %), and EGFR (4 %), but the frequency of these alterations—as indicated—is rather low and several other tumor driving genes have even lower incidence rates (i.e., FOXA1 3 %, BRAF 2 %, and PI3KCA 2 %). Although TP53 and PTEN have been described in advanced disease and poorer survival outcomes, it is not surprising that the majority of these alterations lack independent prognostic significance [3–7].

Another important mutation is represented by the TMPRSS2-ERG gene fusion. This fusion is felt to be associated with the early onset of prostate cancer. Many studies have been performed in order to test the association between TMPRSS2-ERG fusion and outcomes, in particular after radical prostatectomy (RP) or after biopsy but most of have failed to define a clinical significance of this fusion [8–12]. Subsequently, increased copy numbers of the fusion gene have been associated with worse survival or allowed classification of the cohort according to TMPRSS2-ERG fusion status and then certain other markers demonstrated significance based on whether fusion was present or absent [13]. ERG gene assays, which measure the fusion of TMPRSS2 to ERG in prostate cancer tissues, are underway. This tissue assay aims to identify prostate cancer aggressiveness and may lead to personalization of treatment options.

Moreover, recent reports demonstrated the role of different micro-RNAs in prognostication outcome of high-risk prostate cancer treated with RP [14–16]. Among these, miR-221 was recently reported and externally validated as an independent predictor of cancer-related death of high-risk prostate cancer patients, by partially regulating JAK/STAT signaling pathway and sensitizing prostate cancer cells to interferon-gamma treatment [17•]. These analyses are standing out for several reasons. First, the described single biomarker had a high prediction of cancer-related death (area under the curve (AUC) 90.3 %). Second, these results were confirmed by an external validation in an independent cohort. Third, the reported functional analysis demonstrated the biological function of this biomarker as a tumor suppressor regulating the JAK/STAT signaling pathway and sensitization to interferon-gamma treatment; thus, offering the chance for new treatment modalities in the future.

In summary, a multitude of efforts have been undertaken to identify genomic alterations associated with prostate cancer metastasis and to predict an individual’s prognosis. But despite these achievements, the prognostic significance of the majority of molecular alterations described remains unclear. The reasons are multiple and range from the patient cohorts analyzed—which were often not well-annotated, or included only low- and intermediate-risk patients with a long natural history and limited risk for metastasis and prostate cancer death—to probe sampling of a tumor in which tumor multifocality and heterogeneity are known factors complicating biomarker research [18, 19]. Finally and most importantly, the tests are often not validated in independent patient cohorts.

## Decipher^™^

Decipher^™^ is a genomic test that was co-developed by GenomeDx Biosciences (Vancouver, BC, Canada) and the Mayo Clinic to assess the post RP risk of developing recurrence or metastatic disease of prostate cancer patients. This test is based on the RNA expression of 22 genes involved in cell adhesion, migration, tumor motility, immune system modulation, cell cycle control, and some genes with unknown or non-coding function. This test provides a percentage risk of metastases at 5 years. Some retrospective studies have analyzed the ability of Decipher^™^ in predicting recurrence after RP. Alshalafa et al. analyzed differences in patients that developed biochemical recurrence (BCR), clinical metastases, or were free from any recurrence during follow up, finding that Decipher^™^ can represent a useful tool predicting patients that will develop clinical recurrence after BCR [20]. In this setting, many other reports corroborate the ability of this test to predict metastases after therapy with curative intent [21–25]. In a similar context, Klein et al. reported on 162 patients with higher risk prostate cancer including some patients with positive lymph nodes treated with RP without adjuvant therapies and demonstrated an enhanced ability to predict which patient developed metastases when compared to using routine clinico-pathological parameters [25]. Den et al. focused on patients who underwent RP and were found with pT3 and/or positive margins and received postoperative RT [26]. They reported an AUC in predicting BCR and clinical recurrence of 78 and 80 %, respectively. They concluded that a low genomic classifier score could represent an indicator for expectant management with possibility of delayed RT, while patients with high genomic classifier score should be considered for adjuvant RT. Only one report showed the ability of this test in the prediction of cancer-specific mortality (CSM). Cooperberg et al. described 185 surgically treated high-risk prostate cancer patients and compared the Cancer of the Prostate Risk Assessment score (CAPRA) and the Decipher genomic classifier and were able to show an independent prediction of CSM after RP and stronger association of the genomic classifier over CAPRA in this prediction [27•]. This report is of importance because it demonstrated the added value of this test to a standard clinical classification system.

In summary, this test is a promising new tool to better risk stratify prostate cancer patients after RP. One advantage of this test is the fact that it was tested and validated to predict metastasis and prostate cancer mortality after RP. How the results of this genomic test should be used to guide an individual patient’s treatment is unclear but there is suggestion of a predictive component as to the receipt and timing of postoperative radiation therapy. Prospective clinical trials are necessary to prove the value of this promising test to guide patient treatment after radical prostatectomy and should not only evaluate oncological outcome but also the patient’s quality of life and health care resources.

## OncotypeDX®

OncotypeDX® is a diagnostic test that has been developed by Genomic Health (Redwood City, California). By analyzing the expression of 17 genes (includes 5 housekeeping genes) on biopsies, it has been shown to enhance the ability to predict adverse pathology (upgrading and/or upstaging) at RP in patients diagnosed with NCCN very-low, low, and low-intermediate disease. To date, two studies were published assessing the impact of OncotypeDX® on clinical practice. Klein et al. reported the initial discovery and validation on 608 patients who underwent biopsy and had final pathology (grade and stage) assessed by RP [25]. Thus, they were able to describe the impact of OncotypeDX® in predicting adverse pathology at RP. Cullen et al. used a similar setting and analyzed the biopsies from 431 prostate cancer patients with similar NCCN risk criteria. They confirmed the good results reported by Klein et al. to predict adverse pathology at RP and BCR [28].

In summary, OncotypeDX is a new tool to predict adverse pathology in patients with low- to intermediate-risk prostate cancer. But up to now, only one series reported data on patient’s outcome in terms of biochemical recurrence. Future analysis including survival endpoints are necessary to prove if the OncotypeDX test is helpful to better guide patients for active surveillance or active treatment. However, the main challenges in establishing this test in daily clinical practice will be the endpoint used. It is well known that patients with very low- to low/intermediate-risk prostate cancer have a long natural history with a very low risk of metastasis even without any treatment. So what does it mean for a prostate cancer patient if his tumor is upgraded from a small Gleason score 3 + 3 = 6 tumor to a small 3 + 4 = 7 tumor? Does such an upgrading really change the prognosis? Therefore, care has to be taken when active treatment is recommended which is based on a genomic test using surrogate endpoints instead of survival endpoints. However, it appears most useful in the patient and with the provider when contemplating active surveillance and more information is needed. How this test improves such risk stratification vs. multiparametric MRI is not known or if it is complimentary or not to a MRI.

## Prolaris®

Prolaris® is a diagnostic test that has been developed by Myriad Genetics (Wakara Way, Salt Lake City, UT). This test is based on evaluation of the expression of 31 cell cycle progression genes and 15 housekeeping genes. It was designed to evaluate patients with low-risk prostate cancer who may be candidates for active surveillance as well as patients who are at high risk for metastasis and who may benefit from closer monitoring or additional therapy after curative intended treatment.

Cuzick et al. investigated 349 prostate biopsies evaluating the impact of the cell cycle progression (CCP) score based on the Prolaris® platform on outcomes [29]. Bishoff et al. analyzed 582 patients from Martini Clinic (*n* = 283), Durham Veterans Affairs Medical Center (*n* = 176), and Intermountain Healthcare (*n* = 123) and results showed that the CCP score derived from biopsy samples was associated with adverse outcomes after RP [27•]. Cuzick et al. evaluated the CCP score in a subsequent study in two different cohorts of patients. One cohort was treated with RP in the US and the other cohort was treated conservatively or with TUR-P in the United Kingdom. Both studies demonstrated the ability to predict the outcome of the patients, but the endpoint used was different. For the RP cohort, BCR was used and for the conservatively treated UK group of patients’ time-to-death was the endpoint [30]. Cooperberg et al. externally validated the initial results of the RP cohort published by Cuzick et al. in an independent group of 413 patients and confirmed the ability of the CCP score to predict BCR after RP. In addition, they found that combining the CCP score and the CAPRA score improved the concordance index for both the overall cohort and a low-risk subset [31]. Freedland et al. demonstrated the value of the CCP score to predict the outcome of patients treated with primary radiation therapy [32].

The test is commercialized now in several countries and one of the main questions is what to do with the results of this test. Crawford and coworkers pointed this out in an interesting study analyzing how the results of the CCP score test affected the recommendations for prostate cancer treatment. Physicians reported changes of the intended treatment in 65 % of the cases based on the results of the CCP score [33]. These led to a 49.5 % reduction in surgical interventions and a 29.6 % reduction in radiation therapy. They concluded that based on responses of ordering physicians, the CCP report adds meaningful new information to risk assessment for localized prostate cancer patients. Test results led to changes in treatment with reductions but also increases in interventional treatment that were directionally aligned with prostate cancer risk specified by the test. Although it changed management strategies in the majority of cases, it does not answer the question as to whether it was a “right” decision to change and what exact ramifications happen as the course changed perhaps compared to a similar group where the test was not used.

In summary, the CCP score is a promising new tool to better stratify prostate cancer patients. One of the limitations of this tool is the use of biochemical recurrence as an endpoint in most of the studies. What does the prediction of such an endpoint mean for an individual patient? This may differ significantly. A patient with an early PSA recurrence within 1 year after RP with a short PSA-doubling time below 3 months obviously is at the highest risk for progression and most uro-oncologists will agree that this patient benefits from additional treatment. But another patient with a PSA recurrence of 7 years after RP with a PSA doubling time of 18 months has a completely different prognosis, and treatment can be effectively initiated at the time point of recurrence avoiding long-term treatment side effects. The CCP score evaluation should focus more on hard clinical endpoints. Unfortunately, only two studies reported on survival endpoints. Further prospective clinical trials are necessary in the future to prove this test as a prognostic biomarker and a tool to guide treatment. Although a registry-based study demonstrated a significant rate of changes in treatment recommendations the results of the CCP score should be handled carefully until prospective clinical trials demonstrated that these changes really translate into patient benefit.

## Current Main Challenges for Biomarker and Drug Development in Prostate Cancer

Impressive progress has been made in cataloging genomic alterations in prostate cancer and the development of new prognostic tools. Despite these achievements, the prognostic significance of the majority of the molecular alterations and how these can guide an individual patient’s treatment remains unclear. This is mainly the result of the specific challenges in prostate cancer to establishing these relationships: long natural history, necessity for long-term follow-up and large, well annotated cohorts, issues of sampling and tumor multifocality and difficulties in defining and understanding indolent lesions versus driver lesions associated with metastasis and mortality [18]. The clinical and the molecular heterogeneity of prostate cancer complicate accurate risk stratification and selection of management strategies [19].

However, molecular classification can allow the definition of specific prostate cancer subclasses associated with distinct patterns of genomic abnormalities. Genomic, transcriptomic, and metabolomic analyses reveal that prostate cancer can be sub-classified based on gene expression and single copy number alterations, with some success in predicting aggressive features of disease or impact on prognosis [34–36]. Systematic sequencing studies continue to add data allowing the definition of molecular subclasses based on mutations and copy number alterations. These discoveries raise the opportunity that prostate cancer might soon transition from a poorly understood, clinically heterogeneous disease to a collection of homogeneous subtypes identifiable by molecular criteria, associated with specific alterations, with distinct effects on patient prognosis, amenable to specific management strategies, and perhaps vulnerable to more effective targeted therapies [37•, 38•]. These reports are important because they demonstrated tumor heterogeneity in homogeneous groups of patients with localized intermediate risk PCa and metastatic castration-resistant PCa underlining the urgent need of a more tailored, individualized treatment in these patients.

One of the main challenges in prostate cancer management is how to translate the information gained from prognostic tools and molecular sub-stratification information into clinical practice. Widely metastatic prostate cancer is still a lethal disease and despite some survival gains by adding chemotherapy at an earlier stage of the disease, secondary hormonal treatment, or immunological manipulation, survival time of metastatic patients is still limited [39–43]. A multitude of new targeted drugs have been developed over the last decade, and many of these drugs were tested in prostate cancer. Unfortunately, the majority of the phase III drug trials testing such drugs were negative [44–49]. But are these targeted drugs really not effective in prostate cancer? Or was the trial design just too optimistic in believing that a “one treatment fits all” approach would be successful in prostate cancer? Complex and sophisticated clinical trial designs documenting biological mechanisms of action will be needed in the future. One possible solution are collaborative molecular screening platforms in which patients with specific genotypes are rapidly identified and efficiently sorted to be included in sub-group biomarker driven clinical trials. Such screening platforms are allowing integrated drug/biomarker/drug development solutions, cross validation, and benchmarking of technologies alongside strict quality assurance/quality control criteria.

Providing efficient access for patients to molecularly driven clinical trials is one of the aims of the Screening Patients for Efficient Clinical Trial Access (SPECTA) initiative developed by the European Organisation for Research and Treatment of Cancer (EORTC) [50•]. This report is of importance because it described how new disease-oriented screening platforms can optimize drug access, personalized medicine, and health-care delivery. Such platforms are currently under development for colorectal, lung, melanoma, brain, and prostate cancer within the EORTC network.

## Conclusion

Prostate cancer is a clinically and molecularly heterogeneous disease. Discriminating patients with low risk of progression from those with lethal prostate cancer is mandatory to avoid overtreatment on the one side and to improve survival on the other side. Some new promising prognostic tools are now commercially available but their clinical role in individualizing care is still unclear. New, platform-based clinical trial structures are urgently needed to develop and validate prognostic and predictive biomarkers and provide prostate cancer patients with an effective and cost-efficient access to new drugs in the setting of personalized treatment.
